# Paracrine Action of Mesenchymal Stem Cells Revealed by Single Cell Gene Profiling in Infarcted Murine Hearts

**DOI:** 10.1371/journal.pone.0129164

**Published:** 2015-06-04

**Authors:** Yan Yao, Ji Huang, Yongjian Geng, Haiyan Qian, Fan Wang, Xiaohui Liu, Meisheng Shang, Shaoping Nie, Nian Liu, Xin Du, Jianzeng Dong, Changsheng Ma

**Affiliations:** 1 Department of Cardiology, Beijing Anzhen Hospital, Capital Medical University, Beijing Institute of Heart, Lung and Blood Vessel Diseases, Beijing, P. R. China; 2 Center for Cardiovascular Biology and Atherosclerosis, Department of Internal Medicine, The University of Texas, Health Science Center at Houston, Medical School, Texas Heart Institute, Houston, TX, United States of America; 3 Center for Coronary Heart Disease, Department of Cardiology, State Key Laboratory of Cardiovascular Disease, Fuwai Hospital, National Center for Cardiovascular Diseases, Chinese Academy of Medical Sciences and Peking Union Medical College, Beijing, P. R. China; 4 Department of Biostatistics and Epidemiology, University of Pennsylvania, Philadelphia, PA, United States of America; 5 Emergency and Critical Care Center, Beijing Anzhen Hospital, Capital Medical University, Beijing Institute of Heart, Lung and Blood Vessel Diseases, Beijing, P. R. China; University of Louisville, UNITED STATES

## Abstract

**Background:**

Mesenchymal stem cells (MSCs) have been recently demonstrated as a promising stem cell type to rescue damaged myocardium after acute infarction. One of the most important mechanisms underlying their therapeutic effects is the secretion of paracrine factors. However, the expression profile of paracrine factors of MSCs in infarcted hearts, especially at single cell level, is poorly defined.

**Methods and Results:**

We aimed to depict the transcriptional profile of paracrine factors secreted by MSCs *in vivo*, with particular interest in the comparison between normal and infarcted hearts. Bone marrow mesenchymal stem cells were isolated and injected into mice hearts immediately after infarction surgery. Bioluminescence imaging (BLI) indicated a proportion of cells still alive even up to 10 days post surgery. Paralleled with survived cells, cardiac function was significantly improved after MSC injection compared to that in PBS-injected mice, indicated by MRI and histology. Despite increased number of vessels in MSC-injected hearts, endothelial cells and cardiomyocytes transdifferentiation were not observed in infarcted hearts 5 days after infarction. Furthermore, laser capture microdissection (LCM) followed by high through-put real time PCR was employed in our study, uncovering that the injected MSCs, compared to local cardiomyocytes, displayed elevated levels of secreted factors. To further investigate the regulation of those factors, we performed single cell analysis to dissect the gene expression profile of MSCs at single cell level in infarcted and normal hearts, respectively. Consistent with the *in vivo* observation, a similar regulation pattern of those factors was detected in cultured MSCs under hypoxia.

**Conclusions:**

Our study, for the first time, elucidated gene expression profiles, as well as regulation of paracrine factors, of MSCs at single cell level *in vivo*, indicating that paracrine factors from MSCs account for the improvement of cardiac function after infarction.

## Introduction

Mesenchymal stem cell (MSC) is a promising source for the cell-based treatment of acute myocardial infarction (AMI). Numerous experimental and clinical studies have displayed their encouraging therapeutic effects on the improvement of cardiac function [1–3]. However, the mechanisms underlying the benefits of MSC therapy remain debated.

Previously, it was believed that MSCs were able to differentiate into cardiomyocytes, vascular endothelial cells, and vascular smooth muscle cells to repair the damaged myocardium, which is called the trans-differentiation mechanism [4]. However, many investigators have failed to detect persistent engraftment and differentiation of implanted MSCs *in vivo*, and the number of newly regenerated cardiomyocytes is too small to explain the significant functional improvement of infarcted hearts [5,6].

Recently, the focus has shifted to paracrine-centric mechanisms of the MSC-mediated protective effects, as it has been shown that various types of cytokines, chemokines, and growth factors released by MSCs play important roles in cardiac remodeling, angiogenesis, apoptosis, and survival [7,8]. Despite previous studies reporting the expression and function of several paracrine factors, we still lack a full-scale profiling of the paracrine factors expressed by MSCs and adjacent cardiomyocytes *in vivo*, especially in a spatial-temporal manner. So far, few studies have successfully identified paracrine factors that are beneficial for the MSC-mediated protective effects, due to the technical difficulty to examine the expression and secretion of those paracrine factors *in vivo* [9–10].

Single-cell quantitative reverse transcription-polymerase chain reaction (qRT-PCR) is a useful approach widely applied in stem cell and cancer research. By use of this technique, we are able to assess the differences at transcriptional level between cell populations taken from infarcted zones. In the present study, we profiled the expression of twenty-one paracrine factors from MSCs and adjacent cardiomyocytes in infarcted murine hearts, and examined the effect of infarction and hypoxia challenge on their expression patterns both *in vivo* and *in vitro*. We believe that our studies provide a roadmap to comprehensively understand how MSC exerts its effects on myocardium repair after damage [11].

## Materials and Methods

### Ethics Statement

All animal protocols were approved by the Animal Care and Use Committee of Beijing Anzhen Hospital, Capital Medical University.

### Animals

Mice were purchased from the Institute of Laboratory Animal Sciences, Chinese Academy of Medical Sciences (Beijing, China). AMI was performed on 8–10 week old female NOD SCID mice (25~30g). 6–8 week old male transgenic mice (20~25g) of FVB background with β-actin promoter driving Fluc-eGFP (firefly luciferase- enhanced Green Fluorescent Protein) were used as donors for MSCs isolation.

### Isolation, culture and identification of MSCs

MSCs were isolated and cultured as we described previously [12,13]. Briefly, eGFP^+^/Luc^+^ transgenic mice were euthanized, then the tibia and the femur were removed to get the bone marrow. Mononucleated cells were isolated by ficoll density gradient, washed and resuspended in alpha-MEM (GIBCO, CA, USA) supplemented with 10% fetal bovine serum, streptomycin and penicillin. Afterwards, the obtained cells were cultured at 37°C and 5% CO2. Non-adherent cells were removed daily by medium replacement. When the cultures reached 80% of confluence, cells were recovered by a mixture of trypsin/ EDTA and followed by passages.

The third passage of MSCs were collected, and their purity were subsequently verified by fluorescence-activated cell sorting analysis (FACS). As shown in S1 Fig, those MSCs displayed typical fibroblast-like morphology (S1A Fig), and more than 90% cells expressed specific MSC surface marker CD105 and CD90, while lacking the expression of CD45 and CD34 (S1B Fig). For *in vitro* experiments, cells were exposed to normoxia (20% O2, 5% CO2) or hypoxia (1% O2, 5% CO2) conditions for 48 hours.

### Myocardial infarction model and MSC transplantation

AMI was created in female SCID mice by permanent ligation of left anterior descending coronary artery (LAD). The animals were intraperitoneally anesthetized with sodium pentobarbital (50 mg/kg) and mechanically ventilated with room air by using Minivent 845 (Hugo Sachs Electronics, March, Germany). The heart was exposed through a left-sided minithoracotomy, and the left coronary artery was permanently ligated. Infarction was visually confirmed by observation of blanching of the left ventricular myocardium as well as dyskinesis. Immediately after LAD artery ligation, the mice were randomly allocated to receive intramyocardial injections of phosphate-buffered saline (PBS, 20 μl) or MSCs (1×10^6^, 20 μl) at three sites in the infarct border zone.

### *In vivo* bioluminescence imaging

Bioluminescence imaging analysis was performed at days 1, 4, 7, 10 after cell transplantation to monitor cell survival and engraftment by using IVIS 200 system (Caliper, Hopkinton, MA, USA). The mice were routinely anesthetized and then intraperitoneally injected with 100 μl D-luciferin (200 mg/kg to body weight, dissolved in PBS). 10 minutes after the injection, a series of bioluminescent images were recorded for about 20 minutes. Bioluminescent signals were standardized for exposure time and quantified in units of maximum photons per second per square centimeter per steradian (p/s/cm^2^/sr). The image with the greatest signal intensity, which represented the viable injected cells in infarcted hearts, was used for quantification analysis at each time point.

### Cardiac function assessment and HE staining

Before and after transplantation cardiac function was monitored noninvasively by magnetic resonance imaging (MRI). MRI was performed before operation, and 2, 11 days after operation using a 7.0T Biospec small animal experimental scanner (Bruker Biospin, Billerica, MA, USA). The electrocardiographic gating was optimized with two cardiogram electrodes attached to each animal’s forelimbs with respiratory motion and body temperature monitors (Small Animal Instruments, Stony Brook, NY, USA). A series of short-axis views were measured using a retrospectively gated, T1-weighted FLASH sequence (TE 3 ms, TR 6 ms, field of view 45 mm × 45 mm, slice thickness 1.0 mm, imaging matrix 128 ×192). Continuously acquired imaging data from each slice was reconstructed into 10 cine frames. Planimetry measurements of left ventricular myocardial area were conducted by tracing the epicardial and endocardial borders at end-systole and end-diastole using ParaVision software (Bruker Biospin MRI). Ejection fraction (EF) was calculated as the ratio of (LVEDD–LVESD) to LVEDD.

Mice were euthanized and hearts were harvested at day 11 after surgery. Left ventricle was cut into eight fragments from apex to base, and frozen sections (7 mm thickness, 350 mm apart) were randomly chosen from every fragment. The sections were then subjected to hematoxylin and eosin staining (HE staining). All the mice were euthanized at the end of the study.

### Measurement of angiogenesis

At day 5 after operation, mice were euthanized and the hearts were rapidly excised. Paraffin-embedded tissues were cut in 5 μm cross sections through the left ventricle and mounted on slides. After a brief wash in PBS, heart sections were incubated in a blocking buffer(PBS containing 1% bovine serum albumin and 0.1% Triton X-100) at room temperature for 1 hour, then incubated with rabbit anti-CD31 (Abcam, Cambridge, UK) or mouse anti-Actinin 2 (Sigma-Aldrich, MO, USA) primary antibodies at 4°C overnight, followed by secondary antibodies against rabbit and mouse IgG (Invitrogen, CA, USA) respectively at 37°C for 1 hour, and subsequently counterstained with DAPI. Angiogenesis analysis *in vivo* was determined by the expression of a specific endothelial cell marker, CD31. Capillaries were counted in five randomly chosen high-power fields (HPFs) in every chosen section. The results were expressed as capillaries per HPF. The results were expressed as capillaries per HPF. The value was an average of 8 slides per each mouse, total 8 mice per each group.

### Laser capture micro-dissection and qRT-PCR analysis

Mice were euthanized and hearts were removed at day 5 after transplantation, embedded in optimal cutting temperature, and then frozen in liquid nitrogen. Five cyrosections (7 μm thickness, 350 μm apart) of left ventricle were prepared on polyethylene naphthalate membrane-coated slides (P.A.L.M. Microlaser Technologies AG, Bernried, Germany). Green fluorescence visualized under laser microscopy was used as an indicator for micro-dissection. eGFP^+^ MSCs, para-eGFP^+^ cells, remote cells and cells from PBS injected hearts were independently captured by a LMD6000 system (Leica, Germany). The dissected tissues were placed on the caps of micro-centrifuge tubes with 50μl lysis enhanced buffer. After dissection, tissues were collected by centrifugation. Total RNA were extracted from these tissues, and 0.5 μg RNA was used in reverse transcription–polymerase chain reaction(PCR), followed by real-time PCR (Applied biosystems, CA, USA) to examine the expression level of paracrine factors. In addition, different zones including remote zone, border zone and infarcted zone were digested into single cells by collagenase type I(1 mg/ml, Sigma), then eGFP^+^ cells were collected to determine the distribution of MSCs in infarcted hearts.

### Single cell isolation and qRT-PCR Analysis

SCID mice were routinely euthanized at day 5 after surgery and border zones of hearts were removed and digested into single cells. The single cells were stained by H2-Kd and dyecycle (Invitrogen), and then subjected to FACS. MSCs isolated from donor transgenic mice were H2-Kd negative, whereas recipient SCID mice were H2-Kd positive. In addition, dyecycle staining was capable of distinguishing sing cell or fused cell via DNA content, and 2N was considered as a marker of single cell.

Inventoried TaqMan Assays (20×, Applied Biosystems) were pooled and diluted to a final concentration of 0.2× for each of the 24 probes. Individual cells were directly collected into 10 μl RT-PreAmp MasterMix (5.0 μl CellsDirect 2× Reaction Mix [Invitrogen]; 2.5 μl 0.2× assay pool; 0.5 μl RT/Taq enzyme [CellsDirect qRT-PCR kit, Invitrogen]; 2.0 μl TE buffer). The products were analyzed with Universal PCR Master Mix and inventoried TaqMan gene expression assays in 48.48 Dynamic Arrays on a BioMark System (Fluidigm, CA, USA). CT values were calculated from the system’s software (Fluidigm). The resulting values were then normalized to the endogenous controls by subtracting the average of GAPDH expression levels.

### Cytokines array

MSCs were cultured under normaxia and hypoxia conditions. Afterwards, supernatants were harvested, and applied for cytokines array (membrane cytokine array, Abcam). Then, the membranes were developed, and quantified by Image J.

### LCM and Single-Cell Data Processing

All Ct values of genes were converted into relative expression levels by subtracting the values from the assumed baseline value of 32. The resulting values were then normalized to the endogenous controls by subtracting the average of GAPDH expression levels.

### Statistical Analysis

Data are expressed as mean ± standard deviation (SD). Repeated-measures ANOVA with Bonferroni post hoc testing and non-paired Student *t* test were used where appropriate. The level of statistical significance was defined as *P* <0.05. All data collection and analyses were performed with SPSS 13.0.

## Results

### Successful engraftment of MSC in infarcted hearts

To assess the functional outcome of MSC transplantation, we first injected firefly luciferase-bearing MSCs into the mouse myocardium after LAD ligation. Since cell engraftment is a key step in stem cell therapy in myocardial infarction, bioluminescence imaging (BLI) was applied to monitor the survival of transplanted MSCs. As shown in S2A Fig, although firefly luciferase signal was decreased after injection, we were still able to detect considerable signal even on day 10 (S2A and S2B Fig). This observation suggested successful engraftment of MSCs in infarcted hearts.

### MSC Transplantation improves cardiac function after myocardial infarction

**Fig 1:**
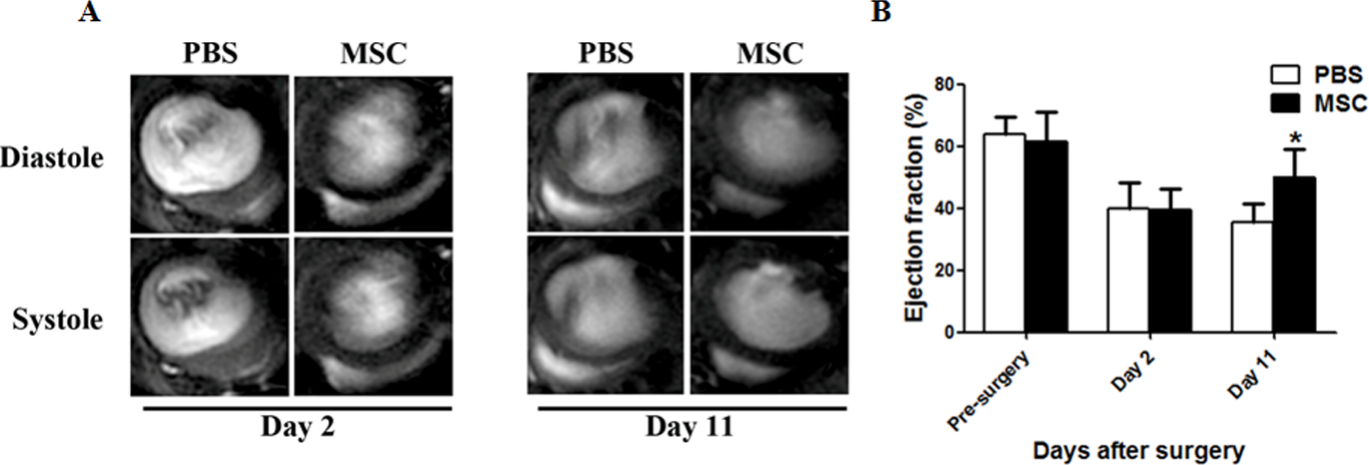
Cardiac functional analysis of infarcted hearts with MSCs transplantation. (A) Representative MRI images of hearts at day 2 and 11 in PBS-injected or MSC-transplanted mice. (B) Quantification of ejection fraction (%) at different time points in two groups mice(n = 8, * *P*<0.01).

To test the therapeutic effects of injected MSCs in infarcted hearts, we performed MRI to evaluate left ventricular ejection fraction of infarcted hearts before and after surgery. The ejection fraction was significantly decreased in both PBS- and MSCs-injected groups on day 2, suggesting successful establishment of the infarction model (Fig 1A and 1B). Interestingly, compared to PBS, MSC transplantation dramatically improved ejection fraction on day 11 (36.73%±4.58% vs. 48.62%±7.25%, *P*<0.01, Fig 1A and 1B). To further confirm the improved outcome of heart function, we performed HE staining to evaluate the ventricular wall thickness 11 days after myocardial infarction. As shown in S3A and S3B Fig, consistent with a functional improvement revealed by MRI, MSC transplantation increased ventricular wall thickness by 58.2% compared to PBS injection only (168.6±91.6 vs. 106.6±57.4 μm, *P*<0.01).

### None of the MSCs differentiates into endothelial cells or cardiomyocytes at early stage of infarction

**Fig 2:**
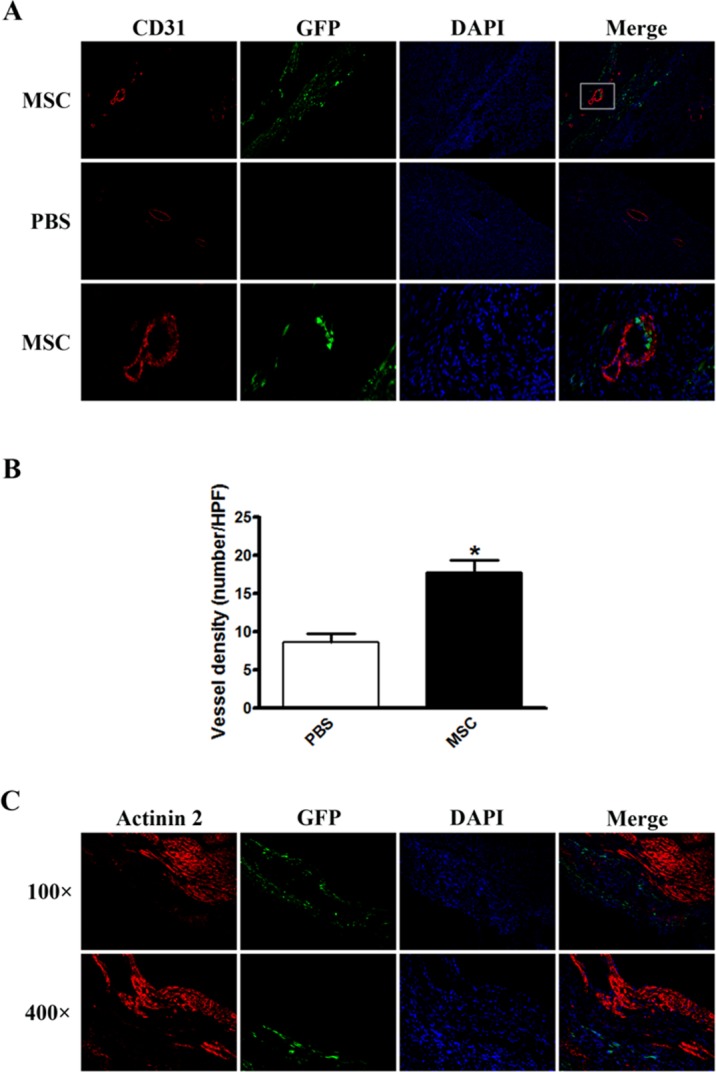
Immunostaining for CD31 and Actinin 2 in infarcted hearts with transplanted MSCs. (A) Representative images of immunostaining against CD31 (red fluorescence) in infarcted hearts. Magnification: 100× in upper and middle panels, 400× in lower panel which is the magnification of the box in upper panel. (B) Statistical analysis of vessel density. The value was an average of 8 slides per each mouse, total 8 mice per each group. (C) Representative images of immunostaining against Actinin 2 (red fluorescence) in infarcted hearts.

To test whether direct cell transdifferentiation from injected MSCs was responsible for the improved cardiac function, we performed immunostaining against CD31, an endothelial cell specific marker, and Actinin 2, a cardiomyocyte specific marker, to determine the transdifferentiation from MSCs to endothelial cells and myocytes, respectively. Compared to the PBS-injected group, MSC injection markedly increased the number of CD31-positive cells, as well as blood vessel density in infarcted hearts 5 days after infarction (17.75±2.6 VS 8.60±1.5/HPF, *P*<0.01, Fig 2A and 2B). However, no colocalization between CD31 and GFP was observed (Fig 2A), indicating no transdifferentiation of endothelial cells from MSCs. Similarly, no transdifferentiation of cardiomyocytes from MSCs was present as none of Actinin 2 and GFP double positive cells were observed (Fig 2C). Taken together, these results suggested mechanisms, other than transdifferentiation, that accounted for the improved cardiac function.

### MSCs express diverse paracrine factors in infarcted hearts

**Fig 3:**
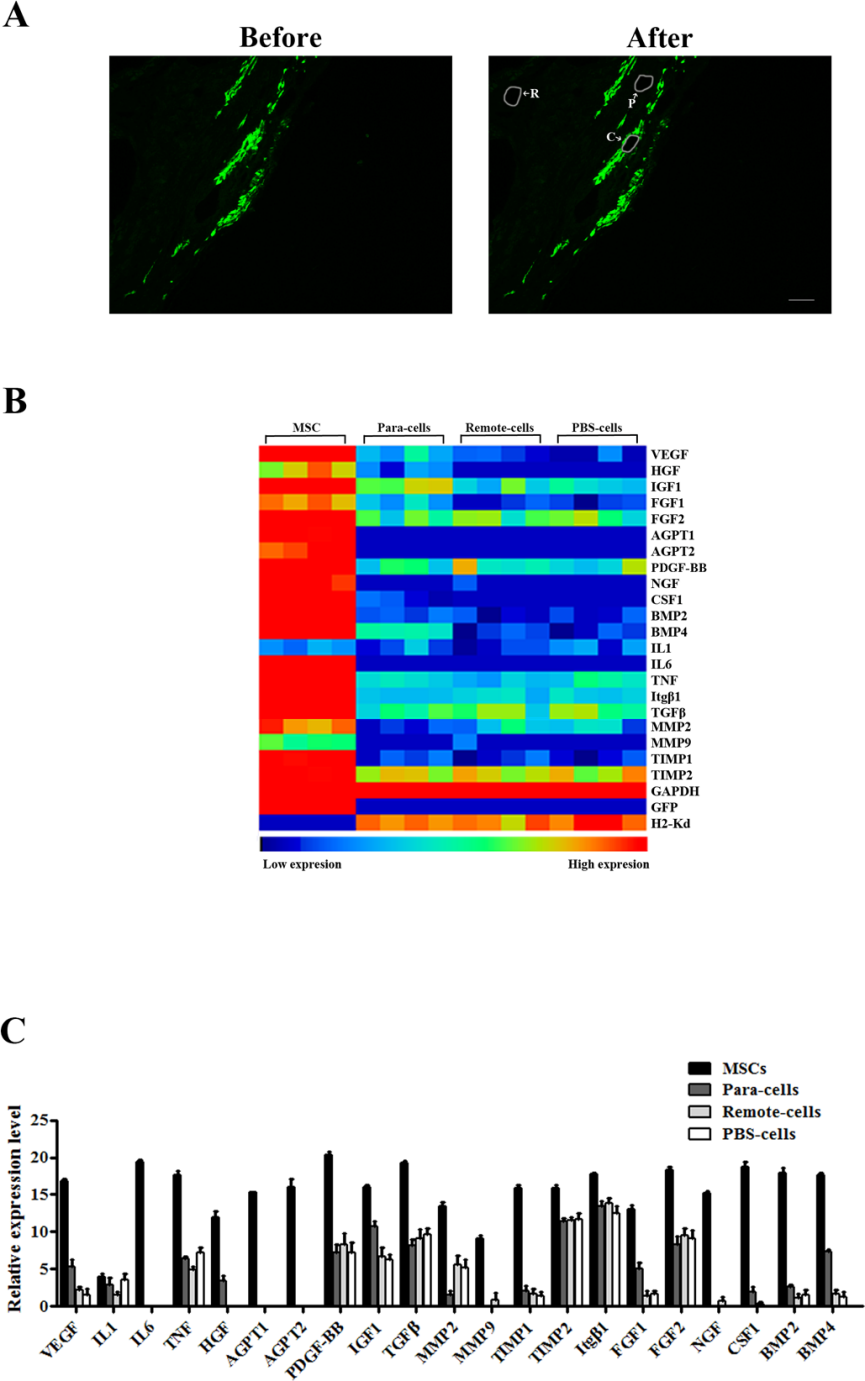
Transcriptomic analysis of gene expression profiles of transplanted MSCs in infarcted hearts. (A) Representative pictures of frozen slides before and after dissection. Selected cells (white arrow) were indicated by white lines (scale bar: 100 μm, magnification 100×). (B) An array displayed overall expression profile of paracrine factors secreted by MSCs, Para-cells, Remote-cells and PBS-cells 5 days after transplantation (n = 8 per group).

Given no transdifferentiation to endothelial cells and cardiomyocytes from injected MSCs, we hypothesized that paracrine effects play a critical role in the improvement of cardiac function. To test our hypothesis, we first explored the distribution of MSCs in infarcted hearts by FACS. Injected cells were enriched in the infarcted zone after surgery. However, these cells quickly diminished. On the contrary, the MSCs percentage was dramatically increased in the remote zone on day 5 post-transplantation, while the cell population was sustained in the border zone (S4 Fig). To further determine how different cell population contributed to paracrine effects in infarcted hearts, we carried out laser capture microdissection (LCM) to collect cell clumps from injected MSCs, local cells adjacent to MSCs (Para-cells), local cells in remote zones (Remote-cells), and local cells from PBS-injected hearts (PBS-cells), respectively (Fig 3A). The expression profiles of paracrine factors from different cell clumps were tested subsequently. As shown in Fig 3B, while IL1 expression levels were comparable among different groups, injected MSCs displayed significantly higher levels of all other factors (VEGF, HGF, IGF1, FGF1, FGF2, AGPT1, AGPT2, PDGF-BB, NGF, CSF1, BMP2, BMP4, IL6, TNF, Itgβ1, TGFβ, MMP2, MMP9, TIMP1, and TIMP2). Interestingly, local cells adjacent to injected MSC exhibited relative higher expression of certain paracrine factors, such as VEGF, HGF, FGF1, CSF1 and BMP4 (Fig 3C). Together, these observations suggested that injected MSCs not only play a predominant role in the expression of paracrine factors, but also affect the paracrine profiles of surrounding cells in infarcted hearts.

### The expression profile of paracrine factors is sensitive to the changes of microenvironment

**Fig 4:**
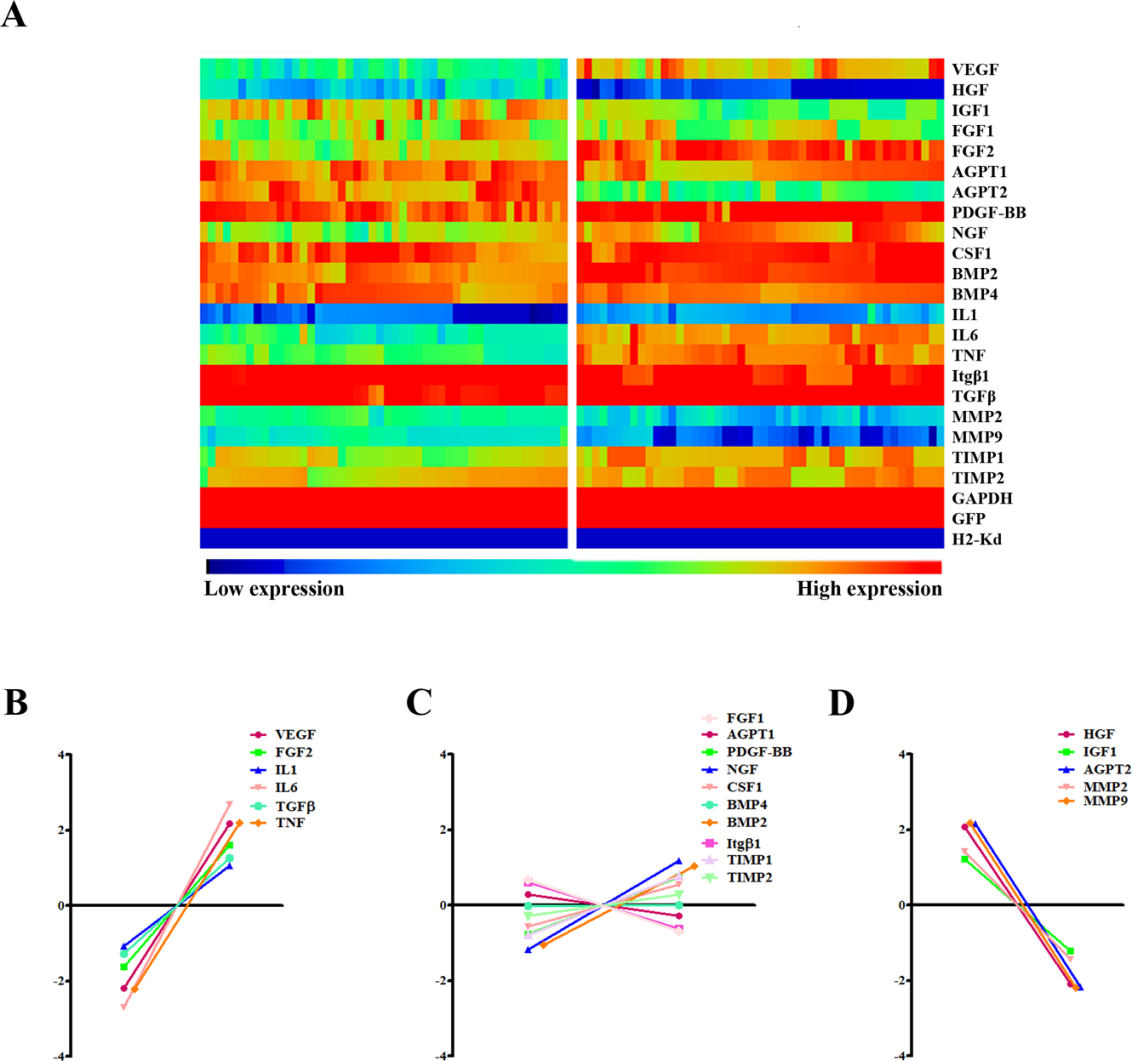
Gene expression profiles in transplanted MSCs of normal and infarcted hearts. (A) An array displayed gene expression profile of different paracrine factors from 48 individual MSCs collected from normal and infarcted hearts, respectively (n = 6 each group). (B-D) Relative expression levels of these paracrine factors (n = 6).

Given a critical role of the microenvironment on cellular behaviors, and due to heterogeneous nature of MSCs, we performed single cell PCR analysis to evaluate expression levels of paracrine factors in infarcted versus normal hearts at the single cell level. Probes against H2-Kd, SRY and eGFP were used to ensure the reliability of isolated MSCs (Fig 4A). Despite the existing variability among single MSCs in one condition, the expression profiles of paracrine factors dramatically changed in infarcted hearts (Fig 4A). While the expression of VEGF, FGF2, IL1, IL6, TGFβ and TNF significantly increased, HGF, IGF1, AGPT2, MMP2 and MMP9 expression decreased under hypoxic stress. The expression of other factors (FGF1, AGPT1, PDGF-BB, NGF, CSF1, BMP4, BMP2, Itgβ1, TIMP1 and TIMP2), however, remained unchanged (Fig 4B–4D).

**Fig 5:**
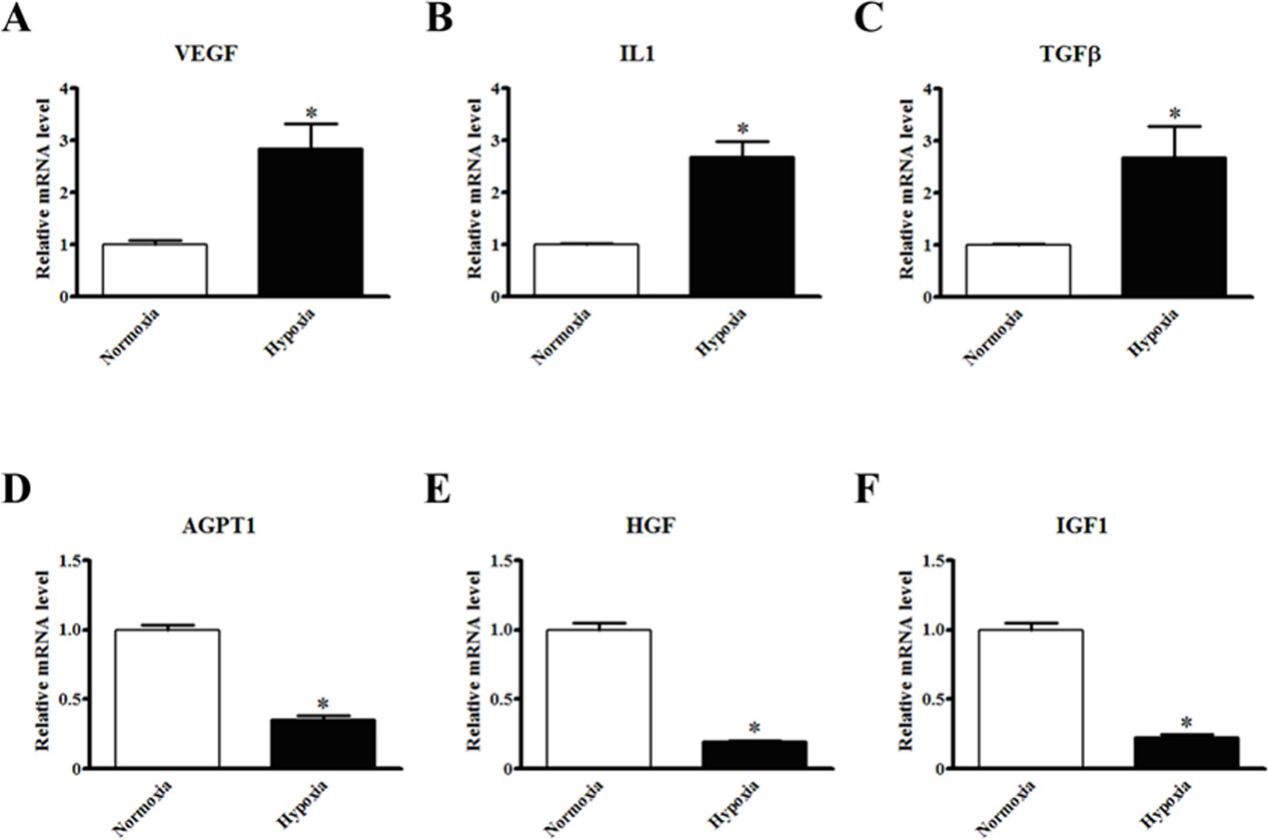
Gene expression profiles of MSCs cultured under hypoxia. Relative expression levels of VEGF (A), IL1 (B), TGFβ (C), AGPT1 (D), HGF (E) and IGF-1 (F) in cultured MSCs with or without hypoxia condition (n = 6).

To verify the regulatory effects of infarction *in vivo*, expression levels of paracrine factors were also assessed in cultured MSCs under hypoxia condition *in vitro*. Similarly, the expression levels of VEGF, IL1 and TGFβ were significantly increased upon the hypoxia exposure (2.84±0.32, 2.68±0.27 and 2.69±0.25 fold vs. normoxia, *P*<0.01), whereas the expression of AGPT1, HGF and IGF1 was decreased (0.35±0.04, 0.19±0.03 and 0.23±0.03 fold vs. normoxia, *P*<0.01), as shown in Fig 5A–5F. In addition to mRNA levels, we detected the released paracrine factors using supernatants collected from MSCs under normxia and hypoxia conditions, respectively. As shown in S5A–S5C Fig, EGF, FGF2, TNFα, TGFα, and EGF secretion was dramatically increased under hypoxia condition. Interestingly, some of these factors were also upregulated at transcriptional level in our mRNA analysis, suggesting the consistency between mRNA synthesis and protein release.

## Discussion

In this study, we systemically profiled the expression of twenty-one paracrine factors from transplanted MSCs and evaluated the effect of infarction on their expression pattern in infarcted murine hearts *in vivo* and *in situ*. Single cell gene profiling revealed that transplanted MSCs expressed considerable paracrine factors *in vivo*. Importantly, we found a significant heterogeneity in the expression of these paracrine factors, as they occurred in a site-dependent manner in the infarcted hearts, with the expression levels increasing sequentially from remote zones to proximal zones to MSCs. Furthermore, we showed that infarction shifted the expression pattern significantly *in vivo*, consistent with the pattern changes caused by hypoxia *in vitro*.

Over the past decades, MSC transplantation has gained much popularity as a potential therapy for AMI and ischemia cardiomyopathy. We and others have demonstrated that MSC treatment can improve cardiac perfusion and contractility in infarcted hearts [12–15]. The mechanism underlying this therapeutic effect has not been clearly defined, with an intense debate over “differentiation” versus “paracrine action”. Like reports from other laboratories [16,17], we failed to demonstrate the differentiation of MSCs into mature cardiac and vascular endothelial cells in the acute phase after infarction, despite enhanced blood vessel density in MSC-transplanted mice compared to PBS-injected mice.

Recently, paracrine action has become the dominant theory explaining the protective effects of MSCs, referring to the release of a wide array of factors that exert their effects on surrounding cells. These factors are involved in the biological processes of angiogenesis, cell proliferation, apoptosis, extracellular matrix remodeling, and inflammation [18,19]. Previous studies have demonstrated MSCs express and secrete a set of paracrine factors, such as FGF2, IGF1, IL1, IL6, TGFβ, TNF, and VEGF, which are increased under hypoxia conditions, while other factors, such as AGPT1 and CSF1, are unchanged (See S2 Table.) [20–23]. Although several studies have suggested critical roles of paracrine effects of MSC transplantation *in vitro*, the overall paracrine factors expressed by MSCs, especially at the single cell level, remained to be systematically determined *in vivo*.

The emergence of single cell analysis technology offers an avenue to discern mechanistic actions of individual cells [24]. By use of single-cell qPCR, we have profiled the expression of twenty-one paracrine factors previously implicated in the pathophysiological processes in infarcted hearts, and demonstrated that the transplanted MSCs express all of these factors *in vivo*. Particularly, the expression of VEGF and IL1 was consistent with previous studies, suggesting the reliability of our analysis [25]. Importantly, we observed that the surrounding cardiomyocytes of MSCs expressed these paracrine factors, indicating that the protective factors were produced not only by transplanted MSCs but also by adjacent cardiomyocytes. We further showed that this effect occurred in a site-dependent manner, as the expression of paracrine effects was more pronounced in the cardiomyocytes adjacent to MSCs than those remote to MSCs. The consequence of such paracrine effects is possibly to generate a cascade of reactions within a short time frame to intensively protect the damaged myocardium. Consistent with this notion, we found that vessel number in MSC-injected hearts was enhanced than PBS-injected hearts at 5 days after infarction. Since it is unlikely that angiogenesis occurred in such short time, we assume that the protective effects of paracrine factors underlie the increased vessel number in MSC-injected hearts. This may partially explain why the small number of survived MSCs can lead to the considerable beneficial effects observed. The initiation of paracrine effect by transplanted MSCs may thus be a critical event in the protection of the injured heart [25].

Given the high sensitivity of MSCs to local microenvironmental influence, we have examined the responses of MSCs to infarction or hypoxia exposure. The expression profile in infarcted hearts is significantly different when compared to that in non-infarcted hearts. Interestingly, factors involved in angiogenesis and cell proliferation were preferentially up-regulated, while those associated with extracellular matrix remodeling were down-regulated. Similar changes in paracrine factor expression under hypoxia have been previously reported *in vitro*. Ohnishi [23] reported that 135 genes were up-regulated under hypoxia, a large number of which were involved in cell proliferation, survival and metabolism. Transplantation of hypoxic preconditioned MSCs resulted in an increase in angiogenesis, as well as enhanced morphologic and functional benefits [26]. Together, these findings suggest that microenvironmental conditions should be taken into consideration in stem cell therapy.

It has been reported that multiple signaling transduction pathways are rapidly activated by MSCs, including PI3K/Akt, Akt/eNOS/Bcl-2, JAK/STAT, ERK and other MAP kinases, thereby promoting survival, proliferation, angiogenesis, anti-apoptosis, extracellular matrix remodeling and inflammation, *etc* [27–30]. Inflammation is one of the causes of up-regulation of VEGF level, a critical factor in cell proliferation, migration, angiogenesis, and cytoprotection [31,32]. IL1 and IL6 can induce VEGF secretion and exert cellular protective function [20,33]. IGF-1 and PDGF have been reported to prevent apoptosis in cardiomyocytes and enhance angiogenesis [34,35]. Furthermore, some chemokines like AGPT1 and HGF are supposed to induce immature stem cells to an injured myocardium and differentiate into large numbers of cardiomyogenic cells *in vivo* and *in vitro* after infarction [20,36]. Involvement of these protective factors in MSC paracrine profiles may underlie overall improvement of cardiac function after AMI [37].

Although we have systemically profiled single cell expression of paracrine factors, and assessed the regulation patterns in infarction, our study presents several limitations. We have not functionally tested which factors are most important for the protective effects, since the study was not designed to establish a causal relationship. Further studies could be done to clarify the roles of each cytokine and evaluate their molecular mechanisms in MSC-mediated paracrine effects.

In summary, our study, for the first time, has employed laser capture micro-dissection and single cell qRT-PCR to systematically profile the expression of paracrine factors in transplanted MSCs in both infarcted and non-infarcted hearts, providing *in vivo* evidence that MSCs exert a paracrine effect on surrounding cardiomyocytes to help improve cardiac function after infarction. These studies will further our understanding on the biological behaviors of MSCs *in vivo*, and shed light on the improvement of MSC therapy in myocardial infarction.

## Supporting Information

S1 ARRIVE Checklist The ARRIVE Guidelines Checklist. Animal Research: Reporting In Vivo Experiments.
(PDF)

S1 Fig Verification of MSCs. (A) Representative images showing typical MSC morphology. (B) FACS to show cultured MSCs displaying MSC specific markers including CD105 and CD90, while lacking the expression of CD45 and CD34.
(TIF)

S2 Fig Bioluminescence imaging of infarcted hearts with MSCs transplantation. (A) Representative pictures of bioluminescence imaging, showing the presence of living MSCs more than 10 days. (B) Quantitative analysis the intensity of bioluminescence signals. Values are the mean of 8 mice per each group.
(TIF)

S3 Fig The ventricular wall thickness with MSC transplantation. (A) Representative images of HE staining 11 days after surgery from two groups. Magnification is 100×. (B) Quantification on histological sections from PBS or MSCs injected hearts. Values are means ± SEM. n = 8 per group. *P<0.05 versus PBS.
(TIF)

S4 Fig The distribution of MSCs in infarcted hearts. At day 2 and 5, FACS was conducted to observe the expression of MSCs at different zones, including remote zone, border zone and infarcted zone.
(TIF)

S5 Fig Cytokines array under normoxia and hypoxia. (A) Blot showing cytokines released from MSCs under normxia and hypoxia conditions. (B) The schematic representing the tested cytokines on the blot. (C) VEGF, FGF2, TNFα, TGFα, and EGF secretion were dramatically increased under hypoxia condition.
(TIF)

S1 Table Primer sequences for RT-PCR. (DOCX)

S2 Table Comparative analysis of expression levels of factors produced by MSCs. (DOCX)
