# Supplementary figures and images for: Paracrine Action of Mesenchymal Stem Cells Revealed by Single Cell Gene Profiling in Infarcted Murine Hearts
Source: PLoS One. 2015 Jun 4;10(6):e0129164. doi: 10.1371/journal.pone.0129164 (PMC4456391; doi:10.1371/journal.pone.0129164)

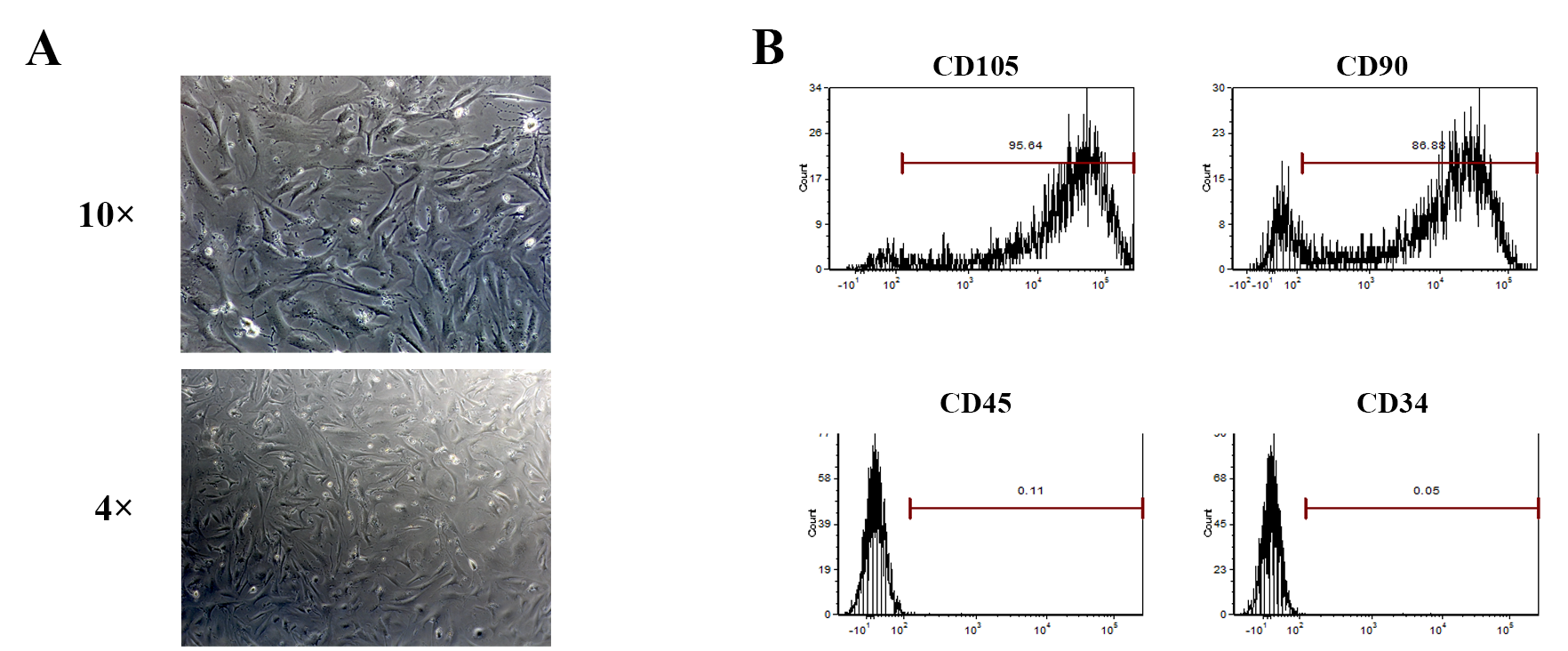

Supplement: S1 Fig — (A) Representative images showing typical MSC morphology. (B) FACS to show cultured MSCs displaying MSC specific markers including CD105 and CD90, while lacking the expression of CD45 and CD34. (TIF) [file pone.0129164.s002.tif]

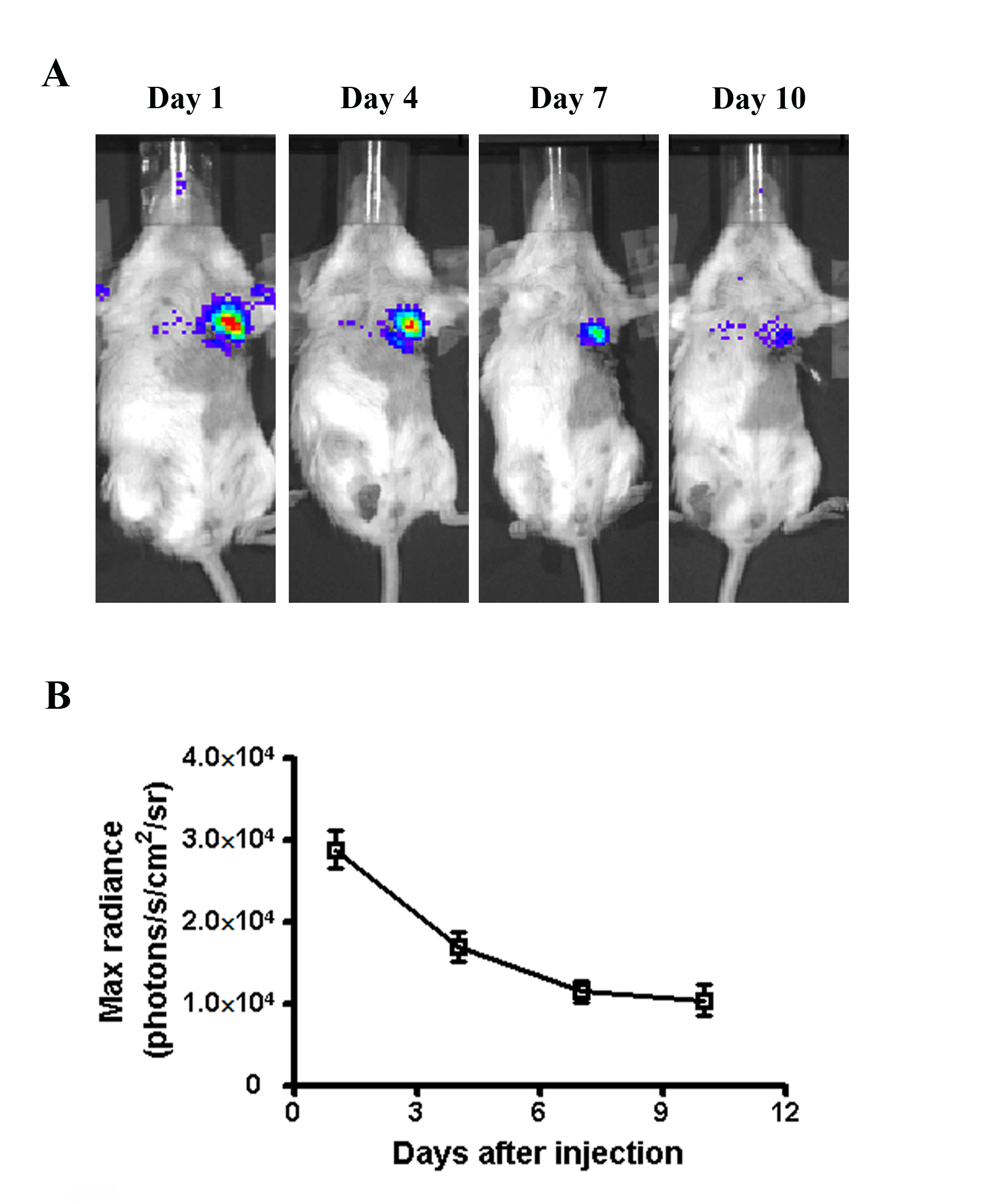

Supplement: S2 Fig — (A) Representative pictures of bioluminescence imaging, showing the presence of living MSCs more than 10 days. (B) Quantitative analysis the intensity of bioluminescence signals. Values are the mean of 8 mice per each group. (TIF) [file pone.0129164.s003.tif]

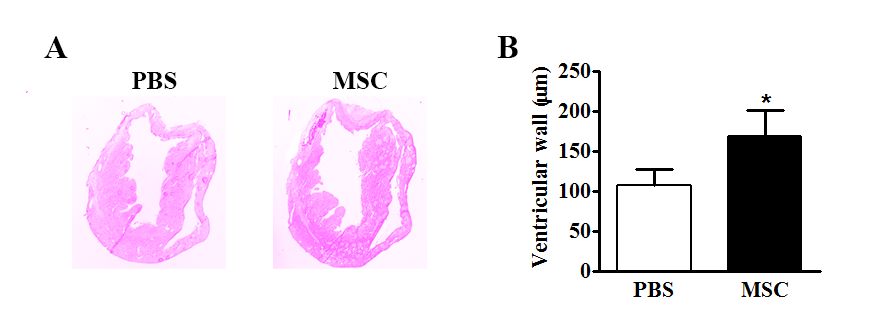

Supplement: S3 Fig — (A) Representative images of HE staining 11 days after surgery from two groups. Magnification is 100×. (B) Quantification on histological sections from PBS or MSCs injected hearts. Values are means ± SEM. n = 8 per group. *P<0.05 versus PBS. (TIF) [file pone.0129164.s004.tif]

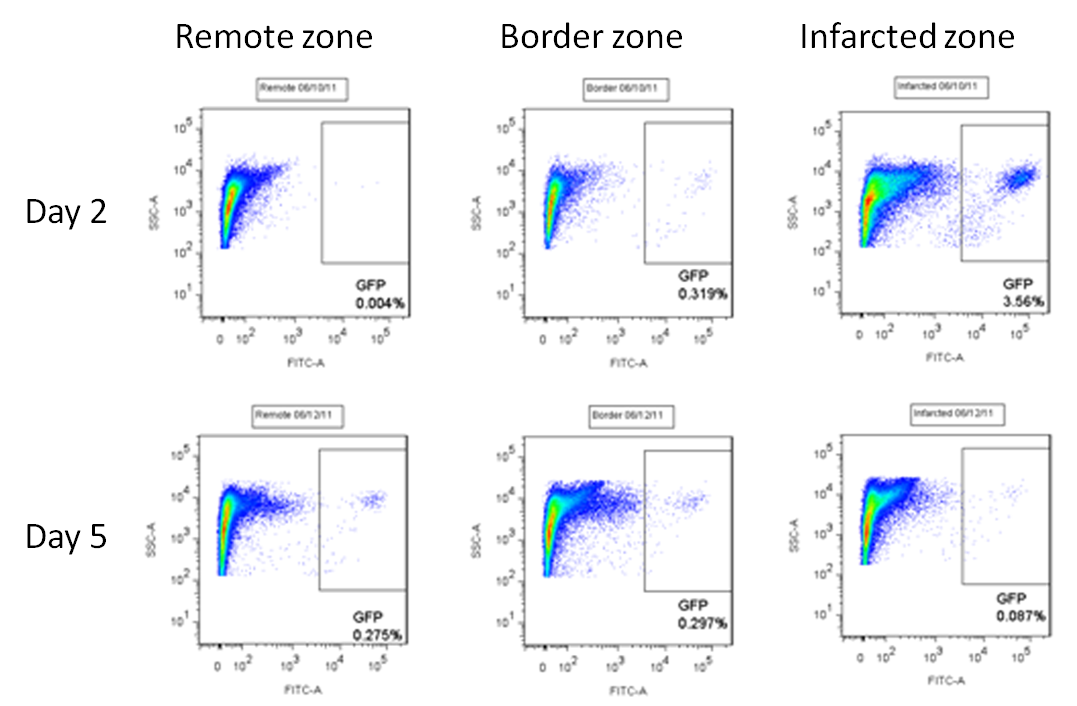

Supplement: S4 Fig — At day 2 and 5, FACS was conducted to observe the expression of MSCs at different zones, including remote zone, border zone and infarcted zone. (TIF) [file pone.0129164.s005.tif]

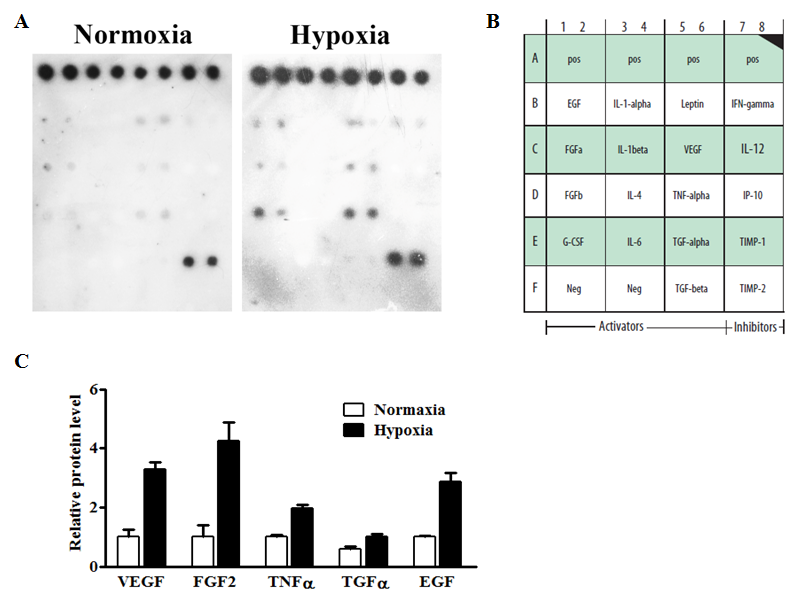

Supplement: S5 Fig — (A) Blot showing cytokines released from MSCs under normxia and hypoxia conditions. (B) The schematic representing the tested cytokines on the blot. (C) VEGF, FGF2, TNFα, TGFα, and EGF secretion were dramatically increased under hypoxia condition. (TIF) [file pone.0129164.s006.tif]
