# Supplementary material for: Paracrine Action of Mesenchymal Stem Cells Revealed by Single Cell Gene Profiling in Infarcted Murine Hearts
Source: PLoS One. 2015 Jun 4;10(6):e0129164. doi: 10.1371/journal.pone.0129164 (PMC4456391; doi:10.1371/journal.pone.0129164)
Supplement: S1 Table — (DOCX) [file pone.0129164.s007.docx]

**Table S1. Primer sequences for RT-PCR**

| Cytokine | Accession | Forward | Reverse |
| --- | --- | --- | --- |
| angiopoietin 1  (AGPT1) | NM_009640 | TACAAAGACGAT  GACGACAAGGAC | AGGGTTAGGGATAG  GCTTACCTTC |
| angiopoietin 2  (AGPT2) | NM_007426 | AGCAGATTT TGG  ATCAGACCAG | GCTCCTTCATGGAC  TGTAGCTG |
| bone morphogenetic  protein 2(BMP2) | NM_007553 | TGAGGATTAGCA  GGTCTTTG | CACAACCATGTCCT  GATAAT |
| bone morphogenetic  protein 4(BMP4) | NM_007554 | CTGGCCCGGAAG  CTAGGTGAGTT | GAGGGCCAGAGACT  GGATCGC |
| colony stimulating  factor 1(CSF1) | NM_007778 | GCCAGCTCCCTGC  CCCAGAGCTTC | GTGTGTCCAAGGTGG  GACCCAACT |
| fibroblast growth  factor 1(FGF1) | NM_010197 | ATGATGACGACG  ACGATGA | CTACGGTTTGGTTTGG  TGTTG |
| fibroblast growth  factor 2(FGF2) | NM_008006 | AGCGACCCACAC  GTCAAACTAC | CAGCCGTCCATCTTCC  TTCATA |
| glyceraldehyde-3-  phosphate dehydrogenase  (GAPDH) | NM_008084 | GCCTCAACGACC  CCTTCAT | ATGTTTGTGATGGGTG  TGAA |
| green fluorescent protein(GFP) | EU056359 | GTTGGAGAAGGTG  GAACCAACTC | AGGGTGTCGCCCTCGAA |
| H2Kd | J00393 | GCCTCAGGACATA  TTAATCTCTGGAG | GCTGATCTCTGAATTCTG  CATGC |
| hepatocyte growth factor(HGF) | NM_001289458 | TAGGAGCCACAA  GGATCTGG | ACATGAAGCAGGAGGA  GGTG |
| insulin-like growth  factor 1（IGF1） | NM_010512 | CAAAAGCAGCC  CGCTCTA | TCGATAGGGACGGGGACT |
| integrin(Itgβ1) | NM_010578 | CCATGCTTGAGAT  AGGAACCAG | TTCAAGTCGGGATGTTTGA  TTT |
| interleukin 1（IL1） | NM_010554 | TTGTGCCAAGTCT  GGAGATG | TTCTCAGAGCGGATGAA  GGT |
| interleukin 6（IL6） | NM_031168 | GCTACCAAACTGG  ATATAATCAGGA | CCAGGTAGCTATGGTACTC  CAGAA |
| matrix metallopeptidase2  (MMP2) | NM_008610 | GCAGATCTCAGGA  GTGACAGG | GATGGCACCCATTTAC  ACCTA |
| matrix metallopeptidase 9(MMP9) | NM_013599 | GCGTCGTGATCCCC  ACTTAC | CAGGCCGAATAGGAGCGTC |
| nerve growth factor(NGF) | NM_013609 | CTTCTTGGGACTG  ATG | CTCATTTCCACGATTT |
| platelet derived  growth factor-BB(PDGF-BB) | NM_011057 | ATCGCCGAGTGC  AAGACGCG | AAGCACCATTGGCCGTC  CGA |
| transforming growth factor(TGFβ) | NM_011577 | CTCCCGTGGCTTC  TAGTGC | GCCTTAGTTTGGACAGG  ATCTG |
| tissue inhibitor of metalloproteinase 1(TIMP1) | NM_001044384 | TACACCCCAGT  CATGGAAAGC | CGGCCCGTGATGAGAAACT |
| tissue inhibitor of metalloproteinase 2(TIMP2) | NM_011594 | GGACCCGAGAA  GACCTCCTT | GCACATCACTCAGAATT  TCAATGG |
| tumor necrosis factor(TNF) | NM_013693 | TTCTCATTCCTG  CTTGTG | TTGGTGGTTTGCTACG |
| vascular endothelial growth factor  (VEGF) | NM_001287056 | TGCACCCACGA  CAGAAGG | GCACACAGGACGGCT  TGA |
