# Supplementary material for: Paracrine Action of Mesenchymal Stem Cells Revealed by Single Cell Gene Profiling in Infarcted Murine Hearts
Source: PLoS One. 2015 Jun 4;10(6):e0129164. doi: 10.1371/journal.pone.0129164 (PMC4456391; doi:10.1371/journal.pone.0129164)
Supplement: S2 Table — (DOCX) [file pone.0129164.s008.docx]

**Table S2.** Comparative analysis of expression levels of factors secreted by MSCs

| Cytokine | Function | Our study | In myocardial infarction animal model in vivo(Ref) | In cultured medium under hypoxia in vitro(Ref) |
| --- | --- | --- | --- | --- |
| AGPT1 | Vascular development, maturation and stabilization | Up-regulated | NA | Unchanged[2] |
| AGPT2 | Vascular development and proliferation | Up-regulated | NA | NA |
| BMP2 | Cell  development and differentiation | Up-regulated | NA | NA |
| BMP4 | Cell  development and differentiation | Up-regulated | NA | NA |
| CSF1 | Monocyte proliferation and differentiation | Up-regulated | NA | Unchanged[2] |
| FGF1 | Endothelial cell proliferation and migration | Up-regulated | NA | NA |
| FGF2 | Endothelial cell and smooth muscle cell proliferation and migration | Up-regulated | NA | Up-regulated[2,3] |
| HGF | Cell growth, angiogenesis, cytoprotection | Up-regulated | NA | Up-regulated or unchanged[2,3] |
| IGF1 | Cell growth and proliferation, anti-apoptosis | Up-regulated | NA | Up-regulated[3,4] |
| Itgβ1 | Cell attachment to the e[xtracellular matrix](http://en.wikipedia.org/wiki/Extracellular_matrix), signal transduction | Up-regulated | NA | NA |
| IL1 | Inflammation regulation, VEGF induction | Unchanged | Up-regulated[1] | Up-regulated[2,4] |
| IL6 | Inflammation regulation, VEGF induction | Up-regulated | NA | Up-regulated[2] |
| MMP2 | [Extracellular matrix](http://en.wikipedia.org/wiki/Extracellular_matrix) remodeling, tubule formation | Up-regulated | NA | Up-regulated or unchanged[2] |
| MMP9 | E[xtracellular matrix](http://en.wikipedia.org/wiki/Extracellular_matrix) remodeling | Up-regulated | NA | Up-regulated or unchanged[2,5] |
| NGF | Cell survival, anti-apoptosis | Up-regulated | NA | NA |
| PDGF-BB | Cell proliferation, angiogenesis | Up-regulated | NA | Up-regulated or unchanged[2,4] |
| TGFβ | Vessel maturation, cell proliferation | Up-regulated | NA | Up-regulated[2] |
| TIMP1 | Cell migration,  [extracellular matrix](http://en.wikipedia.org/wiki/Extracellular_matrix) remodeling | Up-regulated | NA | NA |
| TIMP2 | Cell migration,  [extracellular matrix](http://en.wikipedia.org/wiki/Extracellular_matrix) remodeling | Up-regulated | NA | NA |
| TNF | Cell proliferation, [extracellular matrix](http://en.wikipedia.org/wiki/Extracellular_matrix) remodeling | Up-regulated | NA | Up-regulated[2] |
| VEGF | Cytoprotection, cell proliferation, angiogenesis | Up-regulated | Up-regulated[1] | Up-regulated[2-5] |

NA not available.

**Reference**

1. [Imanishi Y](http://www.ncbi.nlm.nih.gov/pubmed?term=Imanishi%20Y%5BAuthor%5D&cauthor=true&cauthor_uid=18343403), [Saito A](http://www.ncbi.nlm.nih.gov/pubmed?term=Saito%20A%5BAuthor%5D&cauthor=true&cauthor_uid=18343403), [Komoda H](http://www.ncbi.nlm.nih.gov/pubmed?term=Komoda%20H%5BAuthor%5D&cauthor=true&cauthor_uid=18343403), [Kitagawa-Sakakida S](http://www.ncbi.nlm.nih.gov/pubmed?term=Kitagawa-Sakakida%20S%5BAuthor%5D&cauthor=true&cauthor_uid=18343403), [Miyagawa S](http://www.ncbi.nlm.nih.gov/pubmed?term=Miyagawa%20S%5BAuthor%5D&cauthor=true&cauthor_uid=18343403), et al. (2008) Allogenic mesenchymal stem cell transplantation has a therapeutic effect in acute myocardial infarction in rats. [J Mol Cell Cardiol](http://www.ncbi.nlm.nih.gov/pubmed/?term=Allogenic+mesenchymal+stem+cell+transplantation+has+a+therapeutic+effect+in+acute+myocardial+infarction+in+rats) 44:662-671.

2. [Kinnaird T](http://www.ncbi.nlm.nih.gov/pubmed?term=Kinnaird%20T%5BAuthor%5D&cauthor=true&cauthor_uid=14739163), [Stabile E](http://www.ncbi.nlm.nih.gov/pubmed?term=Stabile%20E%5BAuthor%5D&cauthor=true&cauthor_uid=14739163), [Burnett MS](http://www.ncbi.nlm.nih.gov/pubmed?term=Burnett%20MS%5BAuthor%5D&cauthor=true&cauthor_uid=14739163), [Lee CW](http://www.ncbi.nlm.nih.gov/pubmed?term=Lee%20CW%5BAuthor%5D&cauthor=true&cauthor_uid=14739163), [Barr S](http://www.ncbi.nlm.nih.gov/pubmed?term=Barr%20S%5BAuthor%5D&cauthor=true&cauthor_uid=14739163), et al. (2004) Marrow-derived stromal cells express genes encoding a broad spectrum of arteriogenic cytokines and promote in vitro and in vivo arteriogenesis through paracrine mechanisms. [Circ Res](http://www.ncbi.nlm.nih.gov/pubmed/14739163) 94:678-685.

3. [Gnecchi M](http://www.ncbi.nlm.nih.gov/pubmed?term=Gnecchi%20M%5BAuthor%5D&cauthor=true&cauthor_uid=16581974), [He H](http://www.ncbi.nlm.nih.gov/pubmed?term=He%20H%5BAuthor%5D&cauthor=true&cauthor_uid=16581974), [Noiseux N](http://www.ncbi.nlm.nih.gov/pubmed?term=Noiseux%20N%5BAuthor%5D&cauthor=true&cauthor_uid=16581974), [Liang OD](http://www.ncbi.nlm.nih.gov/pubmed?term=Liang%20OD%5BAuthor%5D&cauthor=true&cauthor_uid=16581974), [Zhang L](http://www.ncbi.nlm.nih.gov/pubmed?term=Zhang%20L%5BAuthor%5D&cauthor=true&cauthor_uid=16581974), et al. (2006) Evidence supporting paracrine hypothesis for Akt modified mesenchymal stem cell-mediated cardiac protection and functional improvement. [FASEB J](http://www.ncbi.nlm.nih.gov/pubmed/?term=Evidence+supporting+paracrine+hypothesis+for+Aktmodified+mesenchymal+stem+cell-mediated+cardiac+protection+and+functional+improvement) 20:661-669.

4. [Takahashi M](http://www.ncbi.nlm.nih.gov/pubmed?term=Takahashi%20M%5BAuthor%5D&cauthor=true&cauthor_uid=16603697) , [Li TS](http://www.ncbi.nlm.nih.gov/pubmed?term=Li%20TS%5BAuthor%5D&cauthor=true&cauthor_uid=16603697), [Suzuki R](http://www.ncbi.nlm.nih.gov/pubmed?term=Suzuki%20R%5BAuthor%5D&cauthor=true&cauthor_uid=16603697), [Kobayashi T](http://www.ncbi.nlm.nih.gov/pubmed?term=Kobayashi%20T%5BAuthor%5D&cauthor=true&cauthor_uid=16603697), [Ito H](http://www.ncbi.nlm.nih.gov/pubmed?term=Ito%20H%5BAuthor%5D&cauthor=true&cauthor_uid=16603697),  et al. (2006) Cytokines produced by bone marrow cells can contribute to functional improvement of the infarcted heart by protecting cardiomyocytes from ischemic injury. [Am J Physiol Heart Circ Physiol](http://www.ncbi.nlm.nih.gov/pubmed/?term=Cytokines+produced+by+bone+marrow+cells+can+contribute+to+functional+improvement+of+the+infarcted+heart+by+protecting+cardiomyocytes+from+ischemic+injury) 291:H886-893.

5. Ohnishi S, Yasuda T, Kitamura S, Nagaya N. (2007) Effect of hypoxia on gene expression of bone marrow-derived mesenchymal stem cells and mononuclear cells. Stem Cells 25:1166-1177.

**Non-standard Abbreviations and Acronyms**

AGPT angiopoetin

AMI acute myocardial infarction

BLI bioluminescence imaging

BMP bone morphogenetic protein

CSF colony stimulating factor

DAPI 4,6-diamidino-2-phenylindole

EDTA ethylene diamine tetraacetic acid

EF ejection fraction

eGFP enhanced green fluorescent protein

FACS fluorescence-activated cell sorting

FGF fibroblast growth factor

GAPDH glyceraldehyde phosphate dehydrogenase

HGF hepatocyte growth factor

IGF insulin-like growth factor

IL interleukin

ITG integrin

LAD left anterior descending

LCM laser capture microdissection

Luc luciferase

LVEDD left ventricular end diastolic dimension

LVESD left ventricular end systolic dimension

MMP matrix metalloproteinase

MRI magnetic resonance imaging

MSCs mesenchymal stem cells

NGF nerve growth factor

PBS phosphate-buffered saline

PDGF platelet-derived growth factor

qRT-PCR quantitative reverse transcription-polymerase chain reaction

SCID serious combined immunodeficiency disease

TGF transforming growth factor

TIMP tissue inhibitor of metalloproteinase

TNF tumor necrosis factor

VEGF vascular endothelial growth factor
